# Correction to “A Case of Congenital Afibrinogenemia With Multiple Thrombotic and Hemorrhagic Disorders”

**DOI:** 10.1002/ccr3.9511

**Published:** 2024-10-25

Wei L, Tang Y, Wu Z, Xu P, Mo M. A case of congenital afibrinogenemia with multiple thrombotic and hemorrhagic disorders. *Clin Case Rep*. 2022;10(10):e6395. doi: 10.1002/ccr3.6395. PMID: 36276905; PMCID: PMC9582684.

In Paragraphs 1 and 4 of the Funding Information section, the texts “General Project of Basic and Applied Basic Research of Guangzhou Bureau of Science and Technology, Grant/Award Number: 2060206” and “General Project of Basic and Applied Basic Research of Guangzhou Bureau of Science and Technology (2060206)” were incorrect.

This should read: “General Project of Basic and Applied Basic Research of Guangzhou Bureau of Science and Technology, Grant/ Award Number: 202201020418” and “General Project of Basic and Applied Basic Research of Guangzhou Bureau of Science and Technology (202201020418).”

We apologize for this error.

